# Psychopathological consequences related to COVID-19 infection: The most relevant reactions of the general population

**DOI:** 10.1192/j.eurpsy.2021.695

**Published:** 2021-08-13

**Authors:** G. Serafini, A. Aguglia, A. Amerio, L. Sher, M. Amore

**Affiliations:** 1 Department Of Neuroscience, Rehabilitation, Ophthalmology, Genetics, Maternal And Child Health (dinogmi), University of Genoa, IRCCS Ospedale Policlinico San Martino, Genoa, Italy, Genoa, Italy; 2 Psychiatry, Mount Sinai Hospital, New York City, United States of America; 3 Department Of Neuroscience, Rehabilitation, Ophthalmology, Genetics, Maternal And Child Health (dinogmi), Departimento di Neuroscienze, Università di Genova, Genoa, Italy

**Keywords:** COVID-19 infection, mental health, preventive strategies, Psychological Distress

## Abstract

**Introduction:**

As a result of the emergence of coronavirus disease 2019 (COVID-19) outbreak caused by acute respiratory syndrome coronavirus 2 (SARS-CoV-2) infection in the Chinese city of Wuhan, a situation of socio-economic crisis and profound psychological distress rapidly occurred worldwide.

**Objectives:**

This work aimed to comprehensively review the current literature about the impact of COVID-19 infection on the mental health in the general population.

**Methods:**

A detailed review has been conducted in order to identify the main psychopatological consequences related to Covid-19 infection in the general population.

**Results:**

Various psychological problems and important consequences in terms of mental health including stress, anxiety, depression, frustration, uncertainty during COVID-19 outbreak emerged progressively. The psychological impact of quarantine related to COVID-19 infection has been additionally documented together with the most relevant psychological reactions in the general population related to COVID-19 outbreak.

**Conclusions:**

The role of risk and protective factors against the potential to develop psychiatric disorders in vulnerable individuals with Covid-19 infection need to be carefully addressed in the clinical practice.

